# Variability in quantitative analysis of atherosclerotic plaque inflammation using ^18^F-FDG PET/CT

**DOI:** 10.1371/journal.pone.0181847

**Published:** 2017-08-11

**Authors:** Karel-Jan D. F. Lensen, Alper M. van Sijl, Alexandre E. Voskuyl, Conny J. van der Laken, Martijn W. Heymans, Emile F. I. Comans, Mike T. Nurmohamed, Yvo M. Smulders, Ronald Boellaard

**Affiliations:** 1 Department of Internal Medicine and Institute of Cardiovascular Research (ICaR-VU) VU University Medical Center, Amsterdam, the Netherlands; 2 Department of Rheumatology, Amsterdam Rheumatology and Immunology Center, VU University Medical Center, Amsterdam, the Netherlands; 3 Department of Epidemiology and Biostatistics and EMGO Institute for Health and Care Research, VU University Medical Center, Amsterdam, the Netherlands; 4 Department of Radiology and Nuclear Medicine, VU University Medical Center, Amsterdam, the Netherlands; Biomedical Research Foundation, UNITED STATES

## Abstract

**Background:**

^18^F-FDG-PET(/CT) is increasingly used in studies aiming at quantifying atherosclerotic plaque inflammation. Considerable methodological variability exists. The effect of data acquisition and image analysis parameters on quantitative uptake measures, such as standardized uptake value (SUV) and target-to-background ratio (TBR) has not been investigated extensively.

**Objective:**

The goal of this study was to explore the effect of several data acquisition and image analysis parameters on quantification of vascular wall ^18^F-FDG uptake measures, in order to increase awareness of potential variability.

**Methods:**

Three whole-body emission scans and a low-dose CT scan were acquired 38, 60 and 90 minutes after injection of ^18^F-FDG in six rheumatoid arthritis patients with high cardiovascular risk profiles.Data acquisition (1 and 2) and image analysis (3, 4 and 5) parameters comprised:1. ^18^F-FDG uptake time, 2. SUV normalisation, 3. drawing regions/volumes of interest (ROI’s/VOI’s) according to: a. hot-spot (HS), b. whole-segment (WS) and c. most-diseased segment (MDS), 4. Background activity, 5. Image matrix/voxel size.Intraclass correlation coefficients (ICC’s) and Bland Altman plots were used to assess agreement between these techniques and between observers. A linear mixed model was used to determine the association between uptake time and continuous outcome variables.

**Results:**

1. Significantly higher TBRmax values were found at 90 minutes (1,57 95%CI 1,35–1,80) compared to 38 minutes (1,30 95%CI 1,21–1,39) (P = 0,024) 2. Normalising SUV for BW, LBM and BSA significantly influences average SUVmax (2,25 (±0,60) vs 1,67 (±0,37) vs 0,058 (±0,013)). 3. Intraclass correlation coefficients were high in all vascular segments when SUVmax HS was compared to SUVmax WS. SUVmax HS was consistently higher than SUVmax MDS in all vascular segments. 4. Blood pool activity significantly decreases in all (venous and arterial) segments over time, but does not differ between segments. 5. Image matrix/voxel size does not influence SUVmax.

**Conclusion:**

Quantitative measures of vascular wall ^18^F-FDG uptake are affected mainly by changes in data acquisition parameters. Standardization of methodology needs to be considered when studying atherosclerosis and/or vasculitis.

## Introduction

^18^F-Fluorodeoxyglucose positron emission tomography (^18^F-FDG PET) is a nuclear imaging modality that is increasingly used for assessment of vascular inflammation in patients with atherosclerosis.[1,2] Its clinical relevance is related to the role of inflammation in atherosclerotic plaque development and instability.[3]

Quantitative characteristics of ^18^F-FDG PET are increasingly recognized as providing a more accurate and less observer-dependent measure of inflammatory atherosclerosis than qualitative assessment of PET images.[4] Maximum Standardized Uptake Value (SUVmax) is regularly used for quantification in PET-atherosclerosis studies.[5] SUV is the decay-corrected tissue concentration of intravenously injected ^18^F-FDG normalised for either body weight, lean body mass or body surface area.[6] In addition to SUV, the target-to-background ratio (TBR), which is the ratio of vascular wall and blood pool SUV, is frequently used.[5] Both SUV and TBR correlate with histologically determined macrophage content in atherosclerotic lesions.[7]

To optimize reliability and comparison of results between studies (and within multicenter studies) standardized quantification of vascular wall ^18^F-FDG uptake is essential.[5,8] Nonetheless, there is large variability in data acquisition and image analysis in ^18^F-FDG PET quantification of vascular inflammation. (S1 Table) For instance, arterial segments studied comprised carotid arteries, (parts of) the aorta and/or all large arteries. Also, regions of interest (ROI’s) are not constructed in a consistent manner and ^18^F-FDG uptake time varies, ranging from 45 to 193 minutes.[9,10]

Both image reconstruction and resolution have been shown to influence quantification of ^18^F-FDG uptake in oncological studies.[11,12] In quantitative assessment of vascular inflammation, however, there is still little (though increasing) knowledge on the effect of variability of data acquisition and image analysis.[13]

The objective of this exploratory study was to investigate the effect of several data acquisition and image analysis parameters on quantification of vascular wall ^18^F-FDG uptake.

## Patients and methods

### Patient selection

Six patients with a history of cardiovascular disease (CVD, e.g. TIA, stroke, myocardial infarction or peripheral vascular disease) or a 10-year risk of fatal CVD >10% according to the SCORE risk estimation table were included.[14] In addition, all had rheumatoid arthritis (RA), which is associated with an additional increased risk of cardiovascular disease.[15] This is a subset of patients from a larger trial in which the effects of tumor necrosis factor blocking agents on vascular inflammation are studied. Male and female RA patients > 50 years of age were included, Patients were excluded if they: were using oral corticosteroids, had active tuberculosis or severe infections/sepsis, plasma glucose > 11 mmol/l at time of ^18^F-FDG-PET scan, had moderate heart failure (NYHA class III/IV) or had cancer with a limited life expectancy (< 12 months)

All patients provided written informed consent. The study was approved by the medical ethical review board of the VU University Medical Center.

### Data acquisition and image analysis

#### Data acquisition

A Philips Gemini TOF PET/CT system was used to perform ^18^F-FDG PET/CT studies (Philips medical systems, Eindhoven, The Netherlands). All patients fasted for at least 6 hours prior to the intravenous injection of ^18^F-FDG. Blood glucose was measured before injection in all patients and did not exceed 8 mmol/l (144 mg/dl).

^18^F-FDG uptake time: To explore the effect of uptake-time on the detection of vascular wall inflammation three whole-body (cranium to mid-femur) emission scans were acquired 38, 60 and 90 minutes after intravenous injection of 3,5 Megabecquerel/kilogram ^18^F-FDG.

Scan duration equalled 2 min/bed position. Immediately following the third emission scan, a low-dose CT-scan (80–120 kV, 20–35 mAs) was performed for attenuation correction and anatomic localization (voxel size 1.17x1.17x4 mm). PET images were reconstructed using a time-of-flight ordered subset expectation maximisation algorithm, as implemented by the vendor, providing images with a matrix size of 144x144 and a voxel size of 4x4x4mm.

SUV normalisation: *s*cans were normalised for body weight (BW), lean body mass (LBM) and body surface area (BSA). We explored the effect of these normalisations on SUVmax values.

#### Image analysis

All images were analysed using the PET image analysis research tool developed at the Department of Radiology & Nuclear Medicine of the VU University Medical Center Amsterdam.

Region of interest (ROI): Regional maximum vascular ^18^F-FDG (a surrogate marker of plaque inflammation) can be assessed using several methods.[9,15,16] Three methods were compared in this study.

First, ROI’s were drawn on each axial slice of low-dose CT images in predefined vascular segments. (S2 Table and S1 Fig) Sagittal and coronal views were used to ensure correct placement. The resulting volume of interest (VOI) was transferred to corresponding PET-images. Subsequently, maximal activity in the VOI (*SUVmax WS*) was calculated after (visually) correcting for potential spill-over from adjacent FDG-avid regions (e.g. esophagus for the descending aorta) and for pre-scan glucose levels. This (time-consuming) method should be highly sensitive to detect the most inflamed region.

Second, ROI’s were drawn directly on the axial slice of the PET-image showing the most intense ^18^F-FDG uptake (after visual examination of PET, CT and fused PET/CT images). (S2 Fig) Subsequently, SUVmax in this ROI was calculated (*SUVmax HS*).

Third, *SUVmax most-diseased segment (MDS)* was derived by including two ROI’s in slices adjacent (one proximal and one distal) to the visually determined hot-spot.

Two observers independently determined SUVmax WS and HS. Agreement between observers and between SUVmax WS, HS and MDS was determined.

Background activity: Background (i.e. blood pool) activity was calculated by drawing ROI’s on at least 3 axial slices in the blood pool of the inferior and superior vena cava and the center of the blood pool of the ascending aorta. SUVmean (i.e. average of all voxels in the resulting VOI) was compared between and within these regions at 60 and 90 minutes.

Voxel size/image matrix: The effect of voxel size and/or image rebinning was studied by comparing images with the original PET-voxel size (in these cases the co-registered CT-images were rebinned to PET-voxel size) with images with CT-voxel size (i.e. PET-images were rebinned to the voxel size of the CT-images, potentially affecting measured SUVmax values).

In summary, data acquisition and image analysis parameters that were considered to potentially influence quantification of vascular wall ^18^F-FDG uptake included:

1. ^18^F-FDG uptake time,
2. SUV normalisation,
3. Region or volume of interest (ROI/VOI) definition,
4. Background activity (used for TBR calculations),
5. Image matrix/voxel size.

### Statistical analysis

Continuous data are presented as mean (±Standard Deviation, SD) after normality was ascertained using frequency histograms and Q-Q-plots. Categorical data are presented as proportions. Agreement between analytical methods and observers was assessed using intraclass correlation coefficients (ICC’s; two-way random, absolute agreement) and Bland-Altman plots (for which limits of agreement were calculated). A linear mixed model was used to determine the relation between uptake time and continuous outcome variables (SUV and TBR), correcting for repeated measures within one subject (^18^F-FDG uptake time (i.e. repeated measurements in time in different subjects) was considered fixed and random as vascular segment (i.e. repeated measurement within 1 patient at 1 given time point) was considered as random effects). Levene’s test for homogeneity was used to compare differences in coefficients of variation (for background activity). Values (SUVmax for voxel size) were transformed (using the natural logarithm) due to an association between average SUVmax and the difference between SUVmax for both voxel sizes. Statistical analyses were performed using SPSS analysis software. (SPSS version 20; SPSS Inc.) Individual patient data (anonymized) can be found as supplemental data. (S1 File)

## Results

Patient characteristics are displayed in Table 1. None of the patients used statins and 4 of 6 patients (67%) used anti-hypertensive medication.

**Table 1:** Patient characteristics.

	*Age (years)*	*Sex*	*BMI (kg/m2)*	*DM*	*Plasma glucose* *(mmol/l)*	*History of CVD*
Patient 1	61	male	26,3	No	4,7	Yes
Patient 2	61	male	31,0	No	5,6	No
Patient 3	81	male	26,0	No	6,0	Yes
Patient 4	69	male	16,9	No	6,6	Yes
Patient 5	59	female	21,7	No	5,2	No
Patient 6	61	female	30,5	Yes	7,9	No

DM = type 2 diabetes mellitus; BMI = body mass index; CVD = cardiovascular disease.

### 1. Uptake time

**Fig 1:**
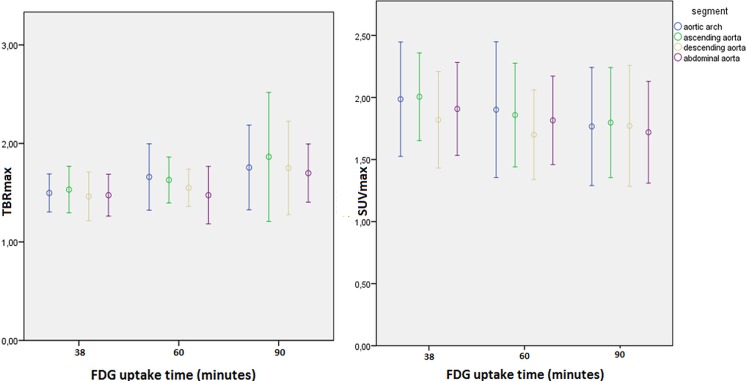
Clustered error plots of TBRmax and SUVmax at all three uptake times in the vascular segments of the aorta.

Results for SUVmax and TBRmax at three post-injection scan times are illustrated in Fig 1. SUVmax gradually decreases over time. In contrast, TBRmax increases. A Significantly higher SUVmax and lower TBRmax values was found at 38 minutes compared to 90 minutes (1.75 vs 1.64, p = 0,025, fixed coefficient -0,115, random intercept 0,091, random slope 0,01) TBRmax, conversely, was significantly higher at 90 minutes compared to 38 minutes (1,57 vs 1,30, p = 0,001, fixed coefficient 0,283, random intercept 0,035, random slope 0,011)

### 2. SUV normalisation

Normalising SUV for body weight, lean body mass and body surface area resulted in an average SUVmax of 2,25 (±0,60), 1,67 (±0,37) and 0,058 (±0,013) respectively which was significantly different between all types of normalisation.

### 3. Region of interest

**Fig 2:**
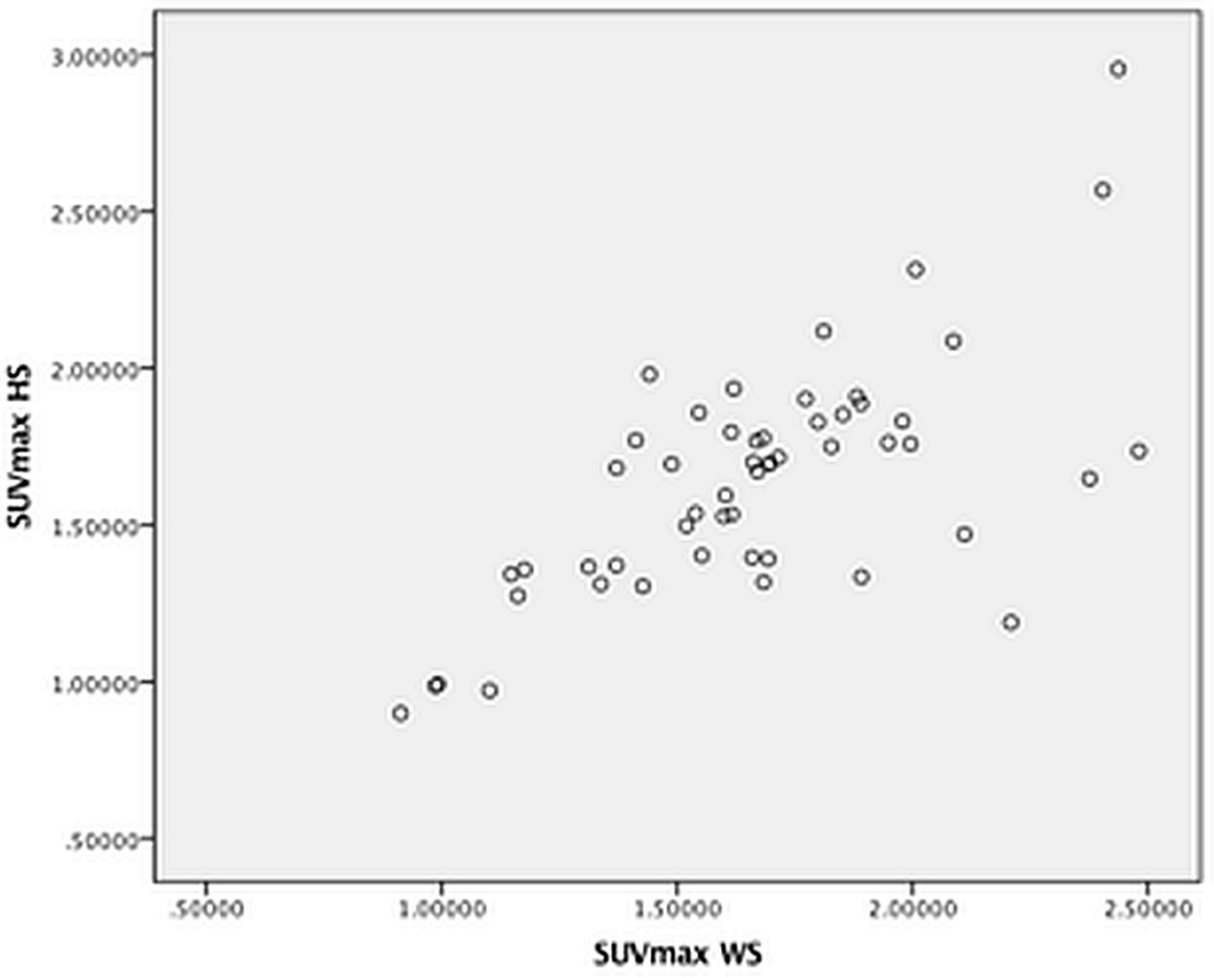
Scatter plot for SUVmax HS versus SUVmax WS. (HS = hot spot, WS = whole segment).

Scatter plots and Bland Altman plots for the agreement between SUVmax HS and WS are displayed in Fig 2 and S3A Fig. Comparable Bland-Altman plots were reconstructed (data not shown) with the average difference between SUVmax WS and HS approaching zero in most vascular segments without many outliers.

Intraclass correlation coefficients were high in all vascular segments when SUVmax HS was compared to SUVmax WS. (Table 2) However, 95% confidence intervals (combined with ICC’s > 0,9) suggest that agreement was best in the aortic segments.

**Table 2:** Intraclass correlation coefficients.

	WS vs HS (95%-CI)	HS vs MDS (95%-CI)	Obs. 1 vs 2 HS	Obs. 1 vs 2 WS
Left carotid	0,73 (0,15–0,92)	0,97 (0,56–0,99)	0,26	0,72
Right carotid	0,73 (0,09–0,92)	0,98 (0,90–1,00)	*	0,95
Ascending aorta	0,92 (0,79–0,97)	0,99 (0,53–1,00)	0,64	0,85
Aortic arch	0,91 (0,77–0,97)	0,98 (0,80–1,00)	0,81	0,99
Descending aorta	0,90 (0,73–0,96)	0,93 (0,66–0,98)	0,67	0,80
Abdominal aorta	0,96 (0,90–0,99)	0,96 (0,00–1,00)	0,83	0,99
Left iliac	0,82 (0,48–0,94)	0,98 (0,91–1,00)	0,77	0,98
Right iliac	0,92 (0,74–0,97)	0,98 (0,78–1,00)	0,79	0,47
Left femoral	0,74 (0,00–0,92)	0,93 (0,12–0,99)	*	0,94
Right femoral	0,93 (0,79–0,98)	0,99 (0,68–1,00)	0,86	0,97

(WS = whole-segment, HS = hot-spot, MDS = most-diseased segment, CI = confidence interval, Obs. = observer)

* Too few observations to calculate ICC.

SUVmax HS was higher than SUVmax MDS in all vascular segments. This is shown in S3B Fig in a Bland Altman plot for the aortic archOn several occasions SUVmax MDS was higher suggesting that the ‘culprit’ lesion was missed using SUVmax HS. Intraclass correlation coefficients were high in all vascular segments. (Table 2)

### Interobserver agreement

Interobserver agreement was excellent for all vascular segments, except the right iliac artery, for the whole-segment method (Table 2). Using the hot-spot method, agreement was excellent for the aortic arch, abdominal aorta and the right femoral artery.

**Fig 3:**
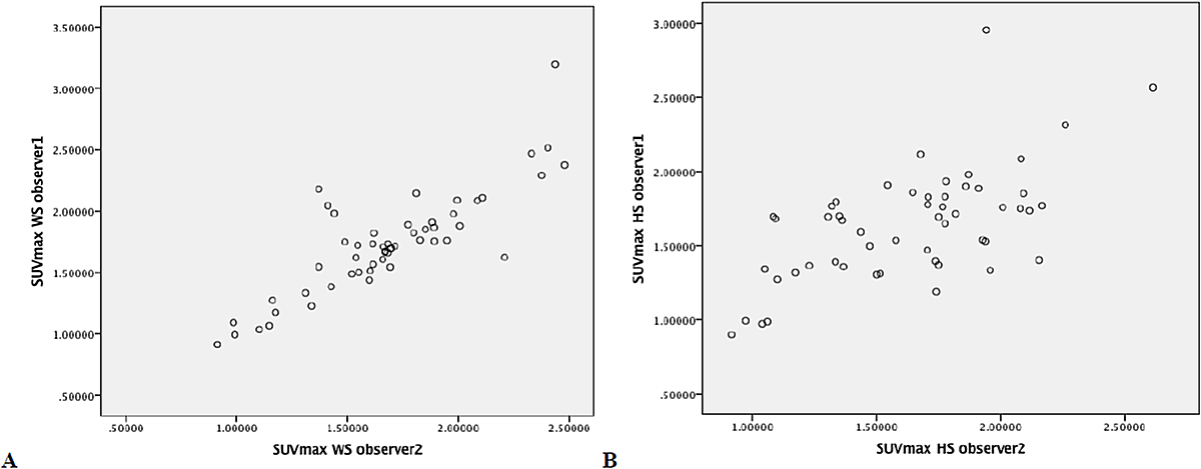
Scatter plots for observer agreement for whole-segment (Figure A.) and Hot-spot (Figure B) method.

Scatter and Bland-Altman plots (Fig 3 and S4 Fig) show excellent agreement and suggest that, for both methods, there were no systematic differences in observer agreement for high or low values, most values lying within 2 standard deviations of the mean difference, indicating good agreement between the observers.

### 4. Background activity

**Fig 4:**
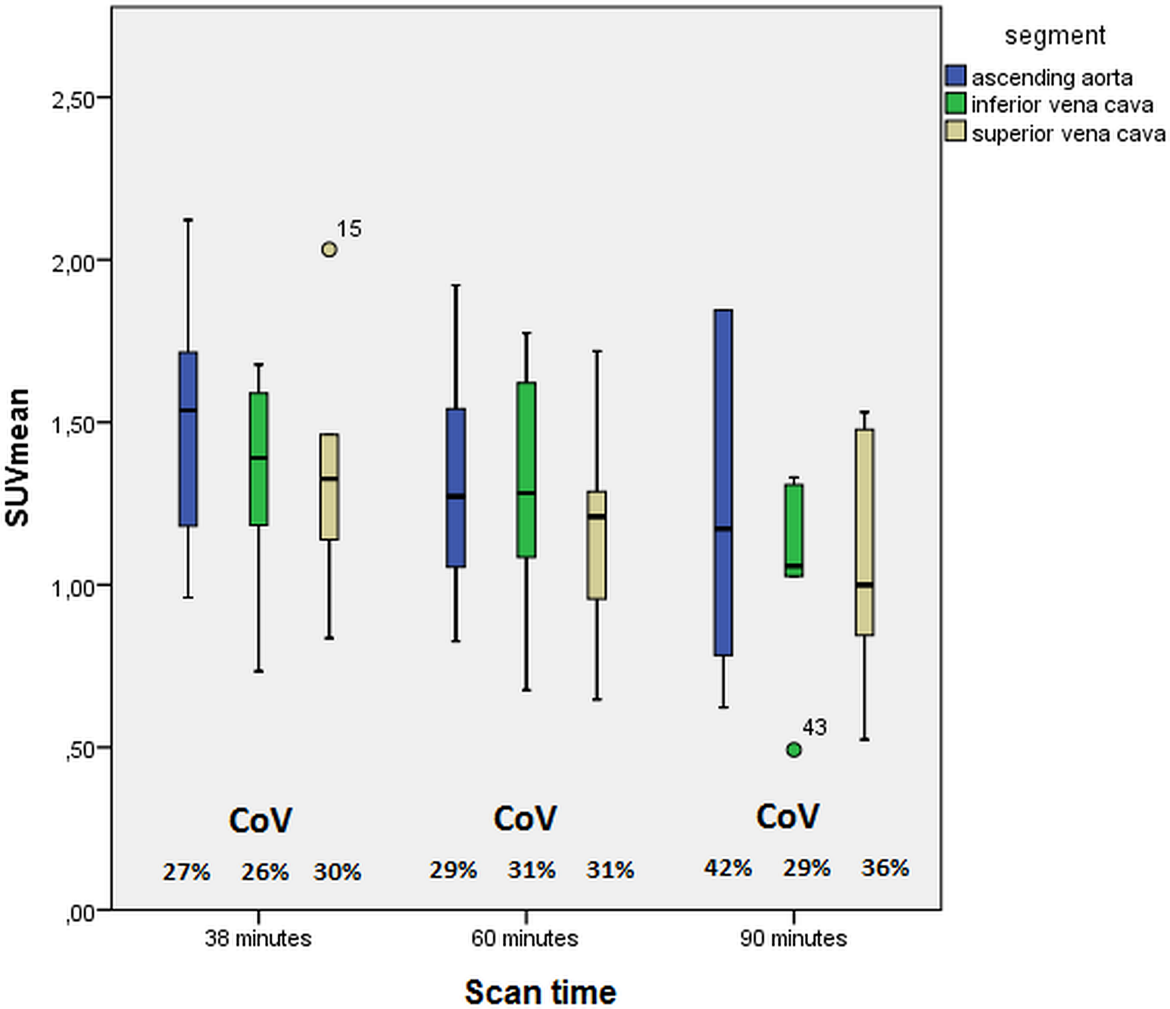
Box plots of blood pool activity expressed as SUVmean in the ascending aorta and inferior and superior vena cava after 38, 60 and 90 minutes of fluorodeoxyglucose (FDG) uptake. (SUVmean = mean standardized uptake value, CoV = coefficient of variation).

Blood pool activity in the 3 vascular segments that were studied at all time points is shown in Fig 4. At 38 minutes, blood pool activity (SUVmean) was significantly higher in the ascending aorta than in the superior vena cava, whereas there were no differences at 60 and 90 minutes. Variation in blood pool activity appears to be larger after 90 minutes, although not statistically significant.

### 5. Voxel size

The average difference between SUVmax PET and CT voxel size approximated zero, SUVmax PET resolution being slightly higher (Bland Altman, S5 Fig). Values were transformed (using the natural logarithm) after which the association between average SUVmax and the difference between SUVmax for both voxel sizes disappeared.

A summary of the main findings is shown in Table 3.

**Table 3:** Summary of main findings.

Acquisition/analysis	Main findings
Data acquisition:	
1. ^18^F-FDG uptake time	- Late imaging decreases vascular SUVmax and increases TBRmax.
2. SUV normalisation	- normalisation significantly affects SUVmax.
Image analysis:	
3. Region of interest	- SUVmax HS comparable to SUVmax WS. - SUVmax HS consistently higher than SUVmax MDS. - Interobserver agreement excellent for WS method. - Interobserver agreement for HS method only for a limited amount of vascular segments (aorta, etc).
4. Background (blood pool) activity	- SUVmean comparable in arterial and venous blood pool and above and below the diaphragm.
5. Image matrix/voxel size	- No effect of image voxel size on SUVmax.

Main findings regarding the possible effect of data acquisition and analytical methods on variability of reported ^18^F-FDG uptake quantification.

## Discussion

Our study shows that data acquisition and image analysis may cause differences in quantification of vascular wall ^18^F-FDG uptake. Data acquisition, in this case ^18^F-FDG uptake time and SUV normalisation appear to affect quantification considerably, whereas image analysis (ROI’s, voxel size and background activity) causes minimal to moderate differences.

Previously, dynamic studies have suggested that late-imaging (i.e. increasing FDG uptake time) is superior for the detection of vessel wall inflammation.[2] This finding has been argued by other investigators.[17] Interestingly, the latter group studied the abdominal aorta as opposed to the carotid arteries. In this study, we showed that SUVmax decreases when uptake time increases, whereas TBRmax increases significantly over time. This effect could not be established for individual segments, most likely due to a small sample size. We may conclude, however, that the decrease in blood pool activity is larger than the decrease in the vascular wall uptake. Furthermore, the inter-subject variability of TBRmax values was larger at late-imaging from which we could hypothesize that vascular wall activity in inflammatory/vulnerable atherosclerotic lesions may decrease at a slower pace than in non-inflammatory lesions. Conversely, late-imaging did not improve correlations between the hot-spot and whole-segment methods. It appears that for the visual detection of atherosclerotic plaque activity late-imaging is not required. This result is in accordance with a previous study in which patterns and locations of ^18^F-FDG uptake could be identified in early and delayed images in patients with carotid artery disease.[10]

Quantification by SUV depends on the type of normalisation used. SUV’s are commonly normalised for either body weight (BW) or lean body mass (LBM), although body surface area (BSA) is also used occasionally. SUVmax BW was clearly higher (i.e. 35%) than SUVmax LBM. SUVmax BSA was much lower due to a different calculation. At present, SUVmax BSA has not been used in vascular wall uptake quantification. In oncology, the most appropriate method for SUV normalization is still a matter of debate, although use of LBM is increasingly being introduced.[18] Our results show that the type of normalisation used should definitely be consistent within (multicenter) studies.

To date, there are no standardized methods to draw regions of interest to detect the most inflamed vascular region. Moreover, a ‘systemic’ approach was initially used in which the average of SUVmax values (as global burden of atherosclerotic disease) of all slices along the course of a vascular segment was calculated.[2] Subsequently, a more ‘focal’ approach, including the single-hottest-slice (hot-spot/HS) and most-diseased segment (MDS), was added.[9,15,16] Several studies have used visually enhanced ^18^F-FDG uptake.[19–21] This method is less time-consuming than a systematic approach in which all axial slices are analysed, however potentially less sensitive to detect the culprit lesion. Our study showed similar SUVmax values for the two methods, indicating that the (visually detected) hot-spot method is equally sensitive and can be used safely without the risk of missing inflamed lesions. Nevertheless, interobserver agreement was not optimal in all regions. Therefore, visual (HS and MDS) analysis should only be used in the aortic arch or abdominal aorta (both showing excellent agreement between HS and WS SUVmax and between observers). These findings contradict previous studies showing high interobserver agreement for all vascular segments.[8,22] In addition, most studies reporting observer agreement show that it is generally good to excellent.[7,23–27] Moderate observer agreement was described in one earlier study that analysed peripheral arteries.[28] Low observer agreement for the visual identification of the site with most intense FDG uptake in peripheral arteries is not surprising as it is more challenging, due to the smaller calibre of the vessel and high FDG uptake in adjacent structures (e.g. muscles) and patient movement, to detect the most inflamed lesion. Studies using a ‘systemic’ approach by averaging SUVmax from multiple axial slices may be less prone to observer variability.

SUVmax MDS was consistently lower than SUVmax HS, probably due to the increasing number of axial slices used to average SUVmax (as it would include more slices having a lower SUVmax value). Though both methods have been previously used, there is no data that supports one of both as predictor of future cardiovascular events.

Background (blood pool) activity is almost exclusively assessed by measuring SUVmean in the blood pool of an adjacent vein (e.g. jugular vein) or the inferior or superior vena cava. Two earlier reports have also calculated blood pool activity in the center of a large artery.[17,29] It has been suggested that spill-over from atherosclerotic lesions may overestimate blood pool activity.[30] Conversely, venous blood pool activity may be lower due to tissue metabolism extracting glucose at the capillary level, yet our results show that arterial and venous blood pool activity were comparable both at 60 and 90 minutes p.i.

To our knowledge, this study is the first to report on the effect of voxel size. We hypothesized that, due to rebinning, conversion of PET-images to CT-voxel size would lead to changes in SUVmax. We were not able to establish this in the vascular segments that we studied. However, comparisons were only made in the large (aortic) segments as visual detection of smaller segments was too difficult after CT images were converted to PET voxel size. Therefore, it remains to be elucidated whether the results also apply to smaller vessels. Nonetheless, our results suggest that adjusting the voxel size of PET-images to CT-voxel size, which is required for proper transfer of VOI’s, can be performed without significantly affecting SUVmax. The purpose of this procedure is to enhance differentiation between vascular segments and structures that may potentially cause spill-over (e.g. oesophagus).

Our study has strengths and limitations. First, our population consists of patients with high cardiovascular risk. RA is associated with increased cardiovascular disease.[15] Additionally, a recent study showed that RA patients had significantly higher SUV’s than a historical control group of patients with cardiovascular disease.[15] As our population had a history of both RA and cardiovascular disease, we were more certain that we were actually studying vascular inflammation, although a gold standard (histological proof) was absent. Whether findings in this patient group can be generalized to atherosclerosis patients without systemic auto-immune inflammation remains to be determined. Second, our multi-segment approach enabled us to study the effects of potential factors on different vascular segments, which proved to be useful as differences between segments in susceptibility to variation were observed.

The small sample size is a limitation. Possibly, statistically significant differences might have been observed, especially for individual segments in the analysis of uptake time variability. Also, the limited number of possible effectors may be considered a limitation.

Parameters that were not investigated, but may potentially influence quantification are reconstruction parameters, pre-scan glucose values (although we did correct for it) and image acquisition parameters (e.g. time/bed position). The European Association of Nuclear Medicine (EANM) has recently published a position statement on the use of ^18^F-FDG PET imaging in atherosclerosis to optimize standardization of arterial PET imaging in which recommendations on these parameters are provided.[5]

Also, ‘atherosclerotic’ lesions on the ^18^F-FDG PET scan were not confirmed by histology or CTA/MRI. Understandably, it was impossible to obtain histological specimens in these patients. In addition, the large amount of radiation of whole-body CTA and costs of whole-body MRI precluded these imaging modalities to be performed. Finally, we only studied the effect of factors potentially influencing focal SUVmax. Earlier studies mainly calculated the average SUVmax in a vascular segment and this method might be less susceptible to the influence of some of the variation in image acquisition and analysis.[31]

## Conclusion

Quantification of vascular inflammation, using SUVmax/TBRmax, is affected by several data acquisition parameters, i.e. ^18^F-FDG uptake time and SUV normalisation. Image analysis may also introduce variability/affect observer agreement. Although the individual factors may not have a large impact by themselves, the cumulative effects of these factors may result in substantial differences in reported SUV’s throughout studies and within multi-center trials. These results stress the need for standardisation in both clinical practice and research settings. Some of our findings corroborate the recently published position statement of the EANM (i.e. recommendation of late-imaging, particularly for TBR measurements) and some add to this statement (importance of standardization of SUV normalisation and preference of visual (i.e. less time-consuming) methods for aortic arch and abdominal aorta.[5]

## Supporting information

S1 Fig In the whole-segment method axial low dose CT slices (Fig A) were used to draw a region of interest (ROI) comprising the entire artery (Fig B). Afterwards this ROI was transferred to the corresponding ^18^F-FDG-PET slices (Fig C,D). Multiple axial ROI’s were drawn resulting in a final volume interest (VOI) which is illustrated in a coronal low-dose CT slice. Maximal standardized uptake values were determined in this VOI after being transferred to the ^18^F-FDG-PET scan.
(TIF)

S2 Fig Axial slice of an ^18^F-FDG-PET scan with a region of interest (ROI) drawn at the site that visually showed most intense ^18^F-FDG uptake. (TIF)

S3 Fig Bland Altman plots for difference between observers for hot-spot (A) and whole-segment (B) method. (SUV = standardized uptake value)
(TIF)

S4 Fig Bland Altman plots for the agreement between the whole-segment and hot-spot method (Fig A) and between hot-spot and most-diseased segment method (Fig B) in the aortic arch. (HS = hot-spot, MDS = most-diseased segment, SUVmax = maximal standardized uptake value).
(TIF)

S5 Fig Bland Altman plots for difference between SUVmax PET and SUVmax CT. (SUV = standardized uptake value, PET = positron emission tomography, CT = computed tomography, Ln = natural logarithm).
(TIF)

S1 Table Variability in data acquisition and image analysis in ^18^F-FDG PET quantification of vascular inflammation in multiple ^18^F-FDG PET studies. (PDF)

S2 Table Definitions of regions of interest (ROI’s). (PDF)

S1 File SPSS file containing anonymized individual patient data. (SAV)

## Abbreviations

^18^F-FDG: ^18^F-Fluorodeoxyglucose
BSA: body surface area
BW: body weight
CT: computed tomography
CVD: cardiovascular disease
EANM: European Association of Nuclear Medicine
HS: hot spot
ICC: intraclass correlation coefficient
LBM: lean body mass
MDS: most diseased segment
PET: positron emission tomography
RA: rheumatoid arthritis
ROI: region of interest
SD: standard deviation
SUV: standardized uptake value
TBR: target to background ratio
VOI: volume of interest
WS: whole segment

## References

[1] Ben-Haim S, Kupzov E, Tamir A, Israel O (2004) Evaluation of 18F-FDG uptake and arterial wall calcifications using 18F-FDG PET/CT. J Nucl Med 45: 1816–1821. 15534049

[2] Rudd JH, Warburton EA, Fryer TD, Jones HA, Clark JC, Antoun N et al (2002) Imaging atherosclerotic plaque inflammation with [18F]-fluorodeoxyglucose positron emission tomography. Circulation 105: 2708–2711. 12057982 10.1161/01.cir.0000020548.60110.76

[3] Libby P (2013) Mechanisms of acute coronary syndromes and their implications for therapy. N Engl J Med 368: 2004–2013. doi: 10.1056/NEJMra1216063 23697515 10.1056/NEJMra1216063

[4] Boellaard R, O'Doherty MJ, Weber WA, Mottaghy FM, Lonsdale MN, Stroobants SG et al (2010) FDG PET and PET/CT: EANM procedure guidelines for tumour PET imaging: version 1.0. Eur J Nucl Med Mol Imaging 37: 181–200. doi: 10.1007/s00259-009-1297-4 19915839 10.1007/s00259-009-1297-4 PMC2791475

[5] Bucerius J, Hyafil F, Verberne HJ, Slart RH, Lindner O, Sciagra R et al (2015) Position paper of the Cardiovascular Committee of the European Association of Nuclear Medicine (EANM) on PET imaging of atherosclerosis. Eur J Nucl Med Mol Imaging. doi: 10.1007/s00259-015-3259-3 26678270 10.1007/s00259-015-3259-3 PMC4764627

[6] Wahl RL, Jacene H, Kasamon Y, Lodge MA (2009) From RECIST to PERCIST: Evolving Considerations for PET response criteria in solid tumors. J Nucl Med 50 Suppl 1: 122S–150S. 50/Suppl_1/122S [pii]; doi: 10.2967/jnumed.108.057307 19403881 10.2967/jnumed.108.057307 PMC2755245

[7] Tawakol A, Migrino RQ, Bashian GG, Bedri S, Vermylen D, Cury RC et al (2006) In vivo 18F-fluorodeoxyglucose positron emission tomography imaging provides a noninvasive measure of carotid plaque inflammation in patients. J Am Coll Cardiol 48: 1818–1824. doi: 10.1016/j.jacc.2006.05.076 17084256 10.1016/j.jacc.2006.05.076

[8] Rudd JH, Myers KS, Bansilal S, Machac J, Pinto CA, Tong C et al (2008) Atherosclerosis inflammation imaging with 18F-FDG PET: carotid, iliac, and femoral uptake reproducibility, quantification methods, and recommendations. J Nucl Med 49: 871–878. doi: 10.2967/jnumed.107.050294 18483100 10.2967/jnumed.107.050294

[9] Bucerius J, Mani V, Moncrieff C, Rudd JH, Machac J, Fuster V et al (2012) Impact of noninsulin-dependent type 2 diabetes on carotid wall (18)f-fluorodeoxyglucose positron emission tomography uptake. J Am Coll Cardiol 59: 2080–2088. doi: 10.1016/j.jacc.2011.11.069 22651864 10.1016/j.jacc.2011.11.069 PMC3392202

[10] Wu YW, Kao HL, Chen MF, Lee BC, Tseng WY, Jeng JS et al (2007) Characterization of plaques using 18F-FDG PET/CT in patients with carotid atherosclerosis and correlation with matrix metalloproteinase-1. J Nucl Med 48: 227–233. 17268019

[11] Huet P, Burg S, Le GD, Hyafil F, Buvat I (2015) Variability and uncertainty of 18F-FDG PET imaging protocols for assessing inflammation in atherosclerosis: suggestions for improvement. J Nucl Med 56: 552–559. jnumed.114.142596 [pii]; doi: 10.2967/jnumed.114.142596 25722452 10.2967/jnumed.114.142596

[12] Boellaard R, Krak NC, Hoekstra OS, Lammertsma AA (2004) Effects of noise, image resolution, and ROI definition on the accuracy of standard uptake values: a simulation study. J Nucl Med 45: 1519–1527. 45/9/1519 [pii]. 15347719

[13] Bucerius J, Mani V, Moncrieff C, Machac J, Fuster V, Farkouh ME et al (2014) Optimizing 18F-FDG PET/CT imaging of vessel wall inflammation: the impact of 18F-FDG circulation time, injected dose, uptake parameters, and fasting blood glucose levels. Eur J Nucl Med Mol Imaging 41: 369–383. doi: 10.1007/s00259-013-2569-6 24271038 10.1007/s00259-013-2569-6 PMC4024166

[14] Conroy RM, Pyorala K, Fitzgerald AP, Sans S, Menotti A, De BG et al(2003) Estimation of ten-year risk of fatal cardiovascular disease in Europe: the SCORE project. Eur Heart J 24: 987–1003. S0195668X03001143 [pii]. 12788299 10.1016/s0195-668x(03)00114-3

[15] Maki-Petaja KM, Elkhawad M, Cheriyan J, Joshi FR, Ostor AJ, Hall FC et al (2012) Anti-tumor necrosis factor-alpha therapy reduces aortic inflammation and stiffness in patients with rheumatoid arthritis. Circulation 126: 2473–2480. CIRCULATIONAHA.112.120410 [pii]; doi: 10.1161/CIRCULATIONAHA.112.120410 23095282 10.1161/CIRCULATIONAHA.112.120410

[16] Fayad ZA, Mani V, Woodward M, Kallend D, Abt M, Burgess T et al(2011) Safety and efficacy of dalcetrapib on atherosclerotic disease using novel non-invasive multimodality imaging (dal-PLAQUE): a randomised clinical trial. Lancet 378: 1547–1559. doi: 10.1016/S0140-6736(11)61383-4 21908036 10.1016/S0140-6736(11)61383-4 PMC4151875

[17] Menezes LJ, Kotze CW, Hutton BF, Endozo R, Dickson JC, Cullum I et al (2009) Vascular inflammation imaging with 18F-FDG PET/CT: when to image? J Nucl Med 50: 854–857. doi: 10.2967/jnumed.108.061432 19443587 10.2967/jnumed.108.061432

[18] Tahari AK, Chien D, Azadi JR, Wahl RL (2014) Optimum lean body formulation for correction of standardized uptake value in PET imaging. J Nucl Med 55: 1481–1484. jnumed.113.136986 [pii]; doi: 10.2967/jnumed.113.136986 24963129 10.2967/jnumed.113.136986 PMC4337860

[19] Figueroa AL, Subramanian SS, Cury RC, Truong QA, Gardecki JA, Tearney GJ et al (2012) Distribution of inflammation within carotid atherosclerotic plaques with high-risk morphological features: a comparison between positron emission tomography activity, plaque morphology, and histopathology. Circ Cardiovasc Imaging 5: 69–77. doi: 10.1161/CIRCIMAGING.110.959478 22038986 10.1161/CIRCIMAGING.110.959478

[20] Lee SJ, On YK, Lee EJ, Choi JY, Kim BT, Lee KH (2008) Reversal of vascular 18F-FDG uptake with plasma high-density lipoprotein elevation by atherogenic risk reduction. J Nucl Med 49: 1277–1282. doi: 10.2967/jnumed.108.052233 18632820 10.2967/jnumed.108.052233

[21] Myers KS, Rudd JH, Hailman EP, Bolognese JA, Burke J, Pinto CA et al (2012) Correlation Between Arterial FDG Uptake and Biomarkers in Peripheral Artery Disease. JACC Cardiovasc Imaging 5: 38–45. doi: 10.1016/j.jcmg.2011.08.019 22239891 10.1016/j.jcmg.2011.08.019 PMC4068152

[22] Rudd JH, Myers KS, Sanz J, Fayad ZA (2007) Multimodality imaging of atherosclerosis (magnetic resonance imaging/computed tomography/positron emission tomography-computed tomography). Top Magn Reson Imaging 18: 379–388. doi: 10.1097/rmr.0b013e3181598db0 18025992 10.1097/rmr.0b013e3181598db0

[23] Choi HY, Kim S, Yang SJ, Yoo HJ, Seo JA, Kim SG et al (2011) Association of adiponectin, resistin, and vascular inflammation: analysis with 18F-fluorodeoxyglucose positron emission tomography. Arterioscler Thromb Vasc Biol 31: 944–949. doi: 10.1161/ATVBAHA.110.220673 21212400 10.1161/ATVBAHA.110.220673

[24] Kim TN, Kim S, Yang SJ, Yoo HJ, Seo JA, Kim SG et al (2010) Vascular inflammation in patients with impaired glucose tolerance and type 2 diabetes: analysis with 18F-fluorodeoxyglucose positron emission tomography. Circ Cardiovasc Imaging 3: 142–148. doi: 10.1161/CIRCIMAGING.109.888909 20061516 10.1161/CIRCIMAGING.109.888909

[25] Tahara N, Kai H, Yamagishi S, Mizoguchi M, Nakaura H, Ishibashi M et al (2007) Vascular inflammation evaluated by [18F]-fluorodeoxyglucose positron emission tomography is associated with the metabolic syndrome. J Am Coll Cardiol 49: 1533–1539. doi: 10.1016/j.jacc.2006.11.046 17418291 10.1016/j.jacc.2006.11.046

[26] Yang SJ, Kim S, Hwang SY, Kim TN, Choi HY, Yoo HJ et al (2012) Association between sRAGE, esRAGE levels and vascular inflammation: analysis with (18)F-fluorodeoxyglucose positron emission tomography. Atherosclerosis 220: 402–406. doi: 10.1016/j.atherosclerosis.2011.11.008 22137663 10.1016/j.atherosclerosis.2011.11.008

[27] Yoo HJ, Kim S, Park MS, Yang SJ, Kim TN, Seo JA et al (2011) Vascular inflammation stratified by C-reactive protein and low-density lipoprotein cholesterol levels: analysis with 18F-FDG PET. J Nucl Med 52: 10–17. doi: 10.2967/jnumed.110.080838 21149476 10.2967/jnumed.110.080838

[28] Kwee RM, Truijman MT, Mess WH, Teule GJ, Ter Berg JW, Franke CL et al (2011) Potential of integrated [18F] fluorodeoxyglucose positron-emission tomography/CT in identifying vulnerable carotid plaques. AJNR Am J Neuroradiol 32: 950–954. doi: 10.3174/ajnr.A2381 21330389 10.3174/ajnr.A2381 PMC7965558

[29] Wasselius J, Larsson S, Jacobsson H (2009) Time-to-time correlation of high-risk atherosclerotic lesions identified with [(18)F]-FDG-PET/CT. Ann Nucl Med 23: 59–64. doi: 10.1007/s12149-008-0207-3 19205839 10.1007/s12149-008-0207-3

[30] Rudd JH, Myers KS, Bansilal S, Machac J, Woodward M, Fuster V et al (2009) Relationships among regional arterial inflammation, calcification, risk factors, and biomarkers: a prospective fluorodeoxyglucose positron-emission tomography/computed tomography imaging study. Circ Cardiovasc Imaging 2: 107–115. doi: 10.1161/CIRCIMAGING.108.811752 19808576 10.1161/CIRCIMAGING.108.811752 PMC3190196

[31] Burger IA, Huser DM, Burger C, von Schulthess GK, Buck A (2012) Repeatability of FDG quantification in tumor imaging: averaged SUVs are superior to SUVmax. Nucl Med Biol 39: 666–670. doi: 10.1016/j.nucmedbio.2011.11.002 22381783 10.1016/j.nucmedbio.2011.11.002

