# Supplementary figures and images for: Variability in quantitative analysis of atherosclerotic plaque inflammation using 18F-FDG PET/CT
Source: PLoS One. 2017 Aug 11;12(8):e0181847. doi: 10.1371/journal.pone.0181847 (PMC5553940; doi:10.1371/journal.pone.0181847)

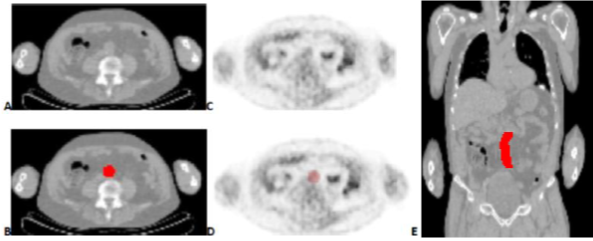

Supplement: S1 Fig — In the whole-segment method axial low dose CT slices (Fig A) were used to draw a region of interest (ROI) comprising the entire artery (Fig B). Afterwards this ROI was transferred to the corresponding 18F-FDG-PET slices (Fig C,D). Multiple axial ROI’s were drawn resulting in a final volume interest (VOI) which is illustrated in a coronal low-dose CT slice. Maximal standardized uptake values were determined in this VOI after being transferred to the 18F-FDG-PET scan. (TIF) [file pone.0181847.s001.tif]

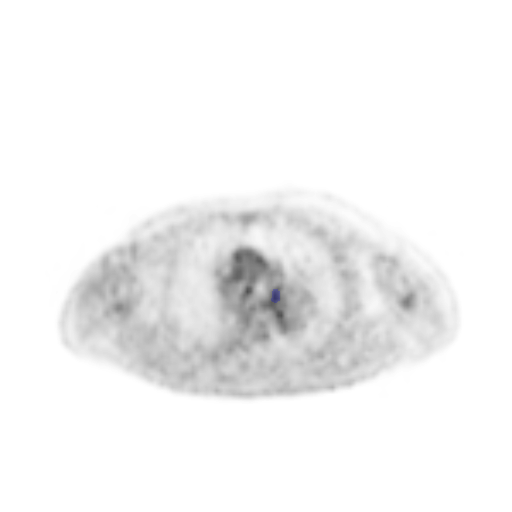

Supplement: S2 Fig — (TIF) [file pone.0181847.s002.tif]

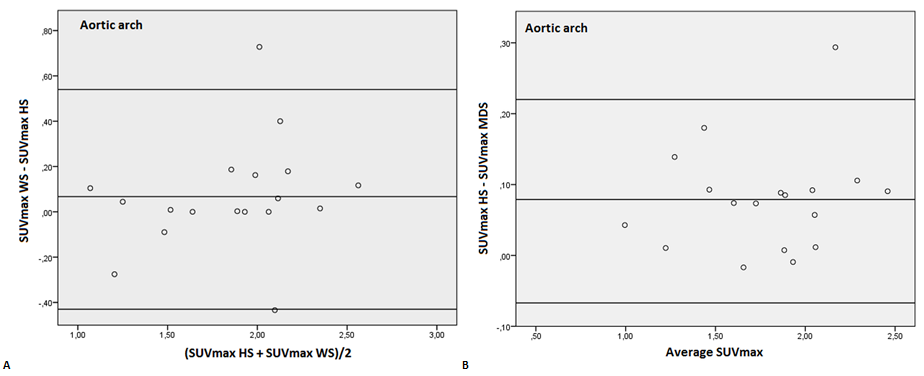

Supplement: S3 Fig — Bland Altman plots for difference between observers for hot-spot (A) and whole-segment (B) method. (SUV = standardized uptake value) (TIF) [file pone.0181847.s003.tif]

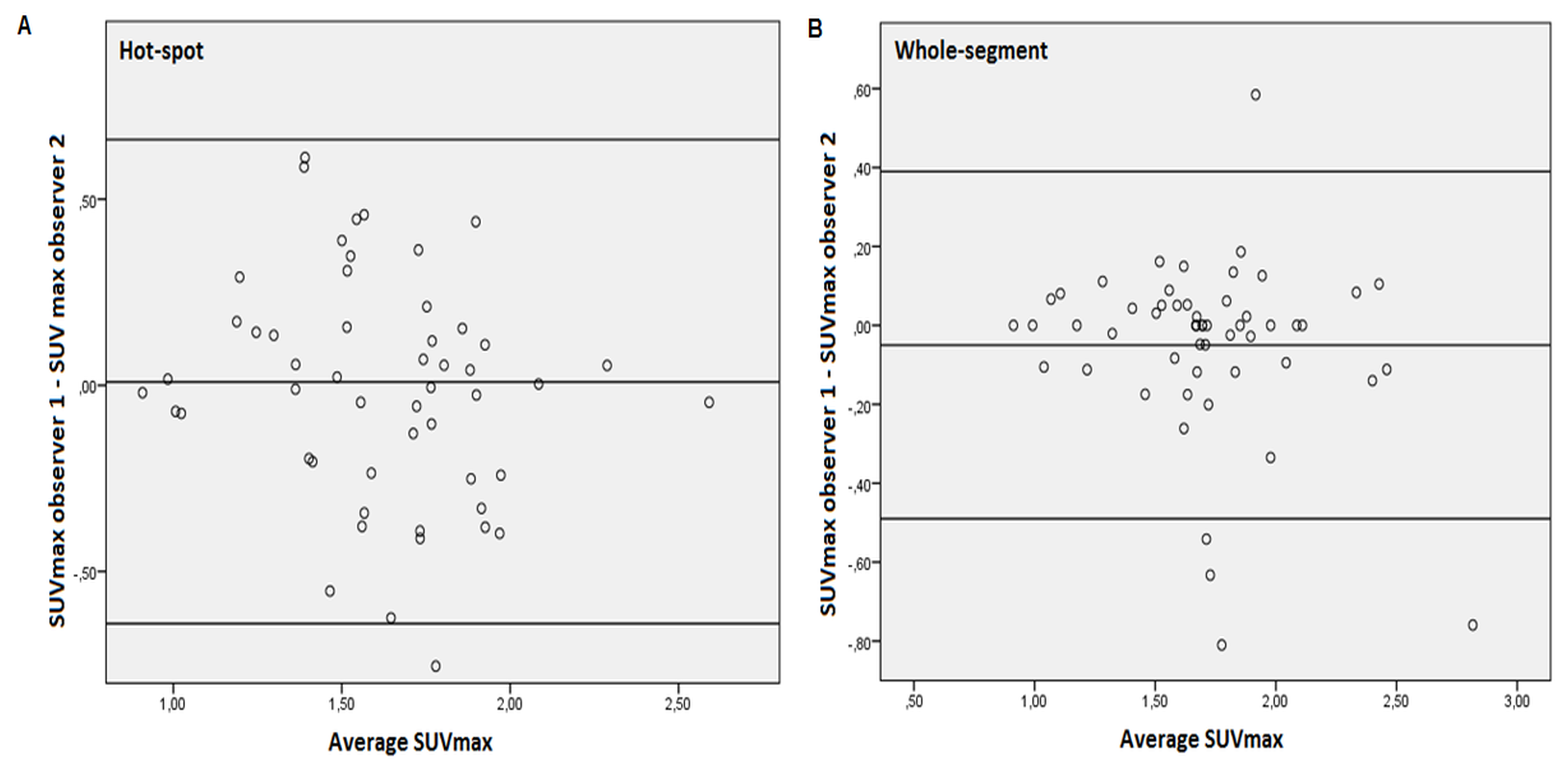

Supplement: S4 Fig — Bland Altman plots for the agreement between the whole-segment and hot-spot method (Fig A) and between hot-spot and most-diseased segment method (Fig B) in the aortic arch. (HS = hot-spot, MDS = most-diseased segment, SUVmax = maximal standardized uptake value). (TIF) [file pone.0181847.s004.tif]

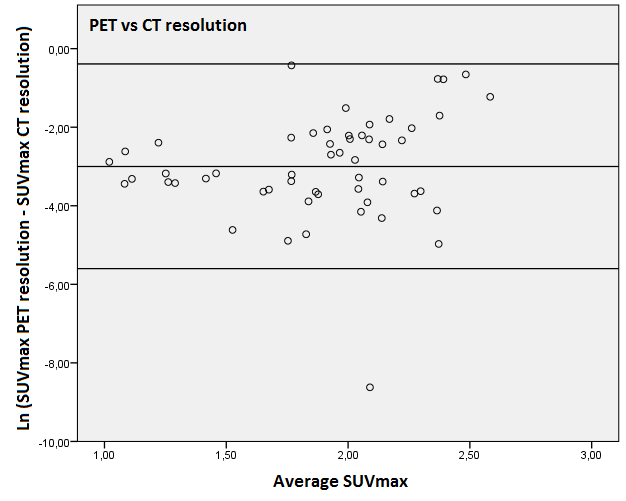

Supplement: S5 Fig — (SUV = standardized uptake value, PET = positron emission tomography, CT = computed tomography, Ln = natural logarithm). (TIF) [file pone.0181847.s005.tif]
