# Supplementary material for: Variability in quantitative analysis of atherosclerotic plaque inflammation using 18F-FDG PET/CT
Source: PLoS One. 2017 Aug 11;12(8):e0181847. doi: 10.1371/journal.pone.0181847 (PMC5553940; doi:10.1371/journal.pone.0181847)
Supplement: S1 Table — (PDF) [file pone.0181847.s006.pdf]

Appendix

Author	SUVmax/mean	Background correction	(clear) description of method of drawing ROI/VOI	Arteries analysed	PI-interval (min)	Sagittal and coronal check of ROI/VOI	Spill-over correction applied	SUV normalisation
**Bissonnette *(1)***	SUVmax and mean ¥	VCI/jugular vein	Yes *	Carotid and ascending aorta	120	N.R.	N.R.	BW
**Maki-Petaja*(2)***	SUVmax ¥	VCS	Yes *	Aorta	90	N.R.	N.R.	N.R
**Bucerius*(3)***	SUVmax ¥	Jugular vein	Yes *	Carotid	97 and 193	N.R.	N.R.	BW
**Myers*(4)***	SUVmax ¥	Nearby vein	Yes ¶	Whole-body	90	Yes	N.R.	N.R.
**Yang*(5)***	SUVmax and mean ¥	Jugular vein	Yes	Right carotid	60	Yes	N.R.	BW
**Figueroa*(6)***	SUVmax and mean	Jugular/subclavian vein	Yes ¶	Carotid	90 and 180	N.R.	N.R.	BW
**Yang*(7)***	N.R.	Jugular vein	Yes	Right carotid	60	Yes	N.R.	BW
**Fifer*(8)***	N.R.	VCS/jugular vein	No	Carotid and ascending aorta	90	N.R.	N.R.	N.R.
**Wu*(9)***	SUVmax ¥	VCI/VCS	Yes	Whole-body	60 and 150	Yes	N.R.	BW
**Mizoguchi *(10)***	SUVmax	VCI	Yes	Carotid and ascending aorta	180	N.R.	N.R.	BW
**Fayad*(11)***	SUVmax and mean	VCS/jugular vein	Yes ¶ *	Carotid and ascending aorta	120	N.R.	N.R.	BW
**Derlin*(12)***	SUVmax	VCI/VCS	Yes ¶	Whole-body	60	Yes	Yes	N.R.
**Ishii*(13)***	SUVmean	VCI/VCS	Yes	Ascending aorta and femoral arteries	60	N.R.	N.R.	N.R.
**Kwee*(14)***	SUVmax and mean	Jugular vein	Yes	Carotid	60	N.R.	N.R.	N.R.
**Choi *(15)***	SUVmean	Jugular vein	No	Carotid	60 and 180	N.R.	N.R.	BW
**Grandpierre *(16)***	SUVmax and mean¥	VCI/jugular vein	Yes	Carotid and aortic arch	60	N.R.	N.R.	N.R.
**Choi, Yoo *(17)*, *(18)***	SUVmax en mean¥	Jugular vein	Yes	Carotid	60	Yes	N.R.	BW
**Yoo *(19)***	SUVmax	Jugular vein	Yes	Carotid	N.R.	N.R.	N.R.	BW
**Coulson *(20)***	SUVmax and mean	VCI/ jugular veiin	Yes	Aorta	90	N.R.	N.R.	BW
**Menezes*(21)***	SUVmax	No	Yes*	Carotid and aorta	60	Yes	N.R.	N.R.
**Pedersen *(22)***	SUVmax and mean	No	Ja	Carotid	180	N.R.	N.R.	BW
**Kim *(23)***	SUVmax and mean	Jugular vein	Yes	Right carotid	60	Yes	N.R.	BW
**Kwee*(24)***	SUVmax and mean	N.R.	No*	Carotid	60	N.R.	N.R.	N.R.
**Rudd *(25)***	SUVmax en mean	VCI/jugular vein	Yes	Whole-body	90	N.R.	N.R.	BW
**Rominger *(26)***	SUVmax $^{\beta}$	VCI/VCS	Yes*	Whole-body	60	Yes	N.R.	N.R.
**Menezes *(27)***	SUVmax	Abdominal aorta	Yes*	Abdominal aorta	45,60,120 and 180	N.R.	N.R.	BW
**Wasselius *(28)***	SUVmean	Abdominal and descending aorta	Yes	Whole-body	60	Yes	N.R.	BW
**Wasselius *(29)***	SUVmean	Abdominal and	Yes	Whole-body	60	Yes	N.R.	N.R.

		descending aorta						
**Potter** ***(30)***	SUVmax¥	No	Yes*	Whole-body	60	N.R.	N.R.	BW
**Graebe** ***(31)***	SUVmax and mean	Jugular vein	Yes	Carotid	180	N.R.	N.R.	BW
**Rudd** ***(32)***	SUVmax and mean ¥	VCI/jugular vein	Yes	Iliac, femoral and carotid arteries	90	N.R.	N.R.	BW
**Paulmier** ***(33)***	SUVmax¥	Lung	Yes¶ *	Whole-body	60	N.R.	N.R.	N.R.
**Bural** ***(34)***	SUVmean¥	No	Yes	Whole-body (not carotid)	60	N.R.	N.R.	N.R.
**Arauz** ***(35)***	N.R.	No	Yes *	Carotid	dynamic	N.R.	N.R.	BW
**Rudd** ***(36)***	SUVmean¥	VCI/jugular vein	Yes	Carotid and thoracic aorta	90	N.R.	N.R.	N.R.
**Tahara** ***(37)*, *(38)***	SUVmax¥	No	Yes	Carotid	60	N.R.	N.R.	BW
**Wu** ***(39)***	SUVmax	No	Yes¶ *	Carotid	45 and 150	Yes	N.R.	LBM
**Tahara** ***(40)***	SUVmax	No	Yes ¶ *	Carotid and thoracic aorta	60	N.R.	N.R.	LBM
**Tawakol** ***(41)***	N.R.	Jugular or subclavian vein	Yes ¶ *	Carotid	60	N.R.	N.R.	BW
**Davies** ***(42)***	FDG uptake value	Normal vessel wall	Yes ¶ *	Carotid	dynamic	N.R.	N.R.	N.R.
**Rudd** ***(43)***	FDG uptake value	No	Yes	Carotid	190 min	N.R.	N.R.	N.R.

Table 1. Overview of studies reporting on vascular inflammation in atherosclerosis showing large variability in the application and reporting of methodology used for the quantification of atherosclerotic plaque inflammation. (N.R.=not reported, VCI=inferior vena cava, VCS= superior vena cava, BW= body weight, LBM= lean body mass, SUV= standardized uptake value) *including reporting of focal FDG uptake. ¶ visually enhanced FDG uptake. ¥: average of all slices. $^{\beta}$: average of all vessel segments.

Reference List

(1) Bissonnette R, Tardif JC, Harel F, Pressacco J, Bolduc C, Guertin MC. Effects of the tumor necrosis factor-alpha antagonist adalimumab on arterial inflammation assessed by positron emission tomography in patients with psoriasis: results of a randomized controlled trial. *Circ Cardiovasc Imaging*. 2013;6(1):83-90.

(2) Maki-Petaja KM, Elkhawad M, Cheriyan J et al. Anti-tumor necrosis factor-alpha therapy reduces aortic inflammation and stiffness in patients with rheumatoid arthritis. *Circulation*. 2012;126(21):2473-2480.

(3) Bucerius J, Mani V, Moncrieff C et al. Impact of noninsulin-dependent type 2 diabetes on carotid wall (18)f-fluorodeoxyglucose positron emission tomography uptake. *J Am Coll Cardiol*. 2012;59(23):2080-2088.

(4) Myers KS, Rudd JH, Hailman EP et al. Correlation Between Arterial FDG Uptake and Biomarkers in Peripheral Artery Disease. *JACC Cardiovasc Imaging*. 2012;5(1):38-45.

(5) Yang SJ, Kim S, Choi HY et al. High-sensitivity C-reactive protein in the low- and intermediate-Framingham risk score groups: analysis with 18F-fluorodeoxyglucose positron emission tomography. *Int J Cardiol*. 2013;163(3):277-281.

(6) Figueroa AL, Subramanian SS, Cury RC et al. Distribution of inflammation within carotid atherosclerotic plaques with high-risk morphological features: a comparison between positron emission tomography activity, plaque morphology, and histopathology. *Circ Cardiovasc Imaging*. 2012;5(1):69-77.

(7) Yang SJ, Kim S, Hwang SY et al. Association between sRAGE, esRAGE levels and vascular inflammation: analysis with (18)F-fluorodeoxyglucose positron emission tomography. *Atherosclerosis*. 2012;220(2):402-406.

(8) Fifer KM, Qadir S, Subramanian S et al. Positron emission tomography measurement of periodontal 18F-fluorodeoxyglucose uptake is associated with histologically determined carotid plaque inflammation. *J Am Coll Cardiol*. 2011;57(8):971-976.

(9) Wu YW, Kao HL, Huang CL et al. The effects of 3-month atorvastatin therapy on arterial inflammation, calcification, abdominal adipose tissue and circulating biomarkers. *Eur J Nucl Med Mol Imaging*. 2012;39(3):399-407.

(10) Mizoguchi M, Tahara N, Tahara A et al. Pioglitazone attenuates atherosclerotic plaque inflammation in patients with impaired glucose tolerance or diabetes a prospective, randomized, comparator-controlled study using serial FDG PET/CT imaging study of carotid artery and ascending aorta. *JACC Cardiovasc Imaging*. 2011;4(10):1110-1118.

(11) Fayad ZA, Mani V, Woodward M et al. Safety and efficacy of dalcetrapib on atherosclerotic disease using novel non-invasive multimodality imaging (dal-PLAQUE): a randomised clinical trial. *Lancet*. 2011;378(9802):1547-1559.

(12) Derlin T, Habermann CR, Hahne JD et al. Quantification of [18F]-FDG uptake in atherosclerotic plaque: impact of renal function. *Ann Nucl Med*. 2011;25(8):586-591.

(13) Ishii H, Nishio M, Takahashi H et al. Comparison of atorvastatin 5 and 20 mg/d for reducing F-18 fluorodeoxyglucose uptake in atherosclerotic plaques on positron emission tomography/computed tomography: a randomized, investigator-blinded, open-label, 6-month study in Japanese adults scheduled for percutaneous coronary intervention. *Clin Ther*. 2010;32(14):2337-2347.

(14) Kwee RM, Truijman MT, Mess WH et al. Potential of integrated [18F] fluorodeoxyglucose positron-emission tomography/CT in identifying vulnerable carotid plaques. *AJNR Am J Neuroradiol*. 2011;32(5):950-954.

(15) Choi YS, Youn HJ, Chung WB et al. Uptake of F-18 FDG and ultrasound analysis of carotid plaque. *J Nucl Cardiol*. 2011;18(2):267-272.

(16) Grandpierre S, Desandes E, Meneroux B et al. Arterial foci of F-18 fluorodeoxyglucose are associated with an enhanced risk of subsequent ischemic stroke in cancer patients: a case-control pilot study. *Clin Nucl Med*. 2011;36(2):85-90.

(17) Choi HY, Kim S, Yang SJ et al. Association of adiponectin, resistin, and vascular inflammation: analysis with 18F-fluorodeoxyglucose positron emission tomography. *Arterioscler Thromb Vasc Biol*. 2011;31(4):944-949.

(18) Yoo HJ, Kim S, Park MS et al. Vascular inflammation stratified by C-reactive protein and low-density lipoprotein cholesterol levels: analysis with 18F-FDG PET. *J Nucl Med*. 2011;52(1):10-17.

(19) Yoo HJ, Kim S, Park MS et al. Serum adipocyte fatty acid-binding protein is associated independently with vascular inflammation: analysis with (18)F-fluorodeoxyglucose positron emission tomography. *J Clin Endocrinol Metab*. 2011;96(3):E488-E492.

(20) Coulson JM, Rudd JH, Duckers JM et al. Excessive aortic inflammation in chronic obstructive pulmonary disease: an 18F-FDG PET pilot study. *J Nucl Med*. 2010;51(9):1357-1360.

(21) Menezes LJ, Kayani I, Ben-Haim S, Hutton B, Ell PJ, Groves AM. What is the natural history of 18F-FDG uptake in arterial atheroma on PET/CT? Implications for imaging the vulnerable plaque. *Atherosclerosis*. 2010;211(1):136-140.

(22) Pedersen SF, Graebe M, Fisker Hag AM, Hojgaard L, Sillesen H, Kjaer A. Gene expression and 18FDG uptake in atherosclerotic carotid plaques. *Nucl Med Commun*. 2010;31(5):423-429.

(23) Kim TN, Kim S, Yang SJ et al. Vascular inflammation in patients with impaired glucose tolerance and type 2 diabetes: analysis with 18F-fluorodeoxyglucose positron emission tomography. *Circ Cardiovasc Imaging*. 2010;3(2):142-148.

(24) Kwee RM, Teule GJ, van Oostenbrugge RJ et al. Multimodality imaging of carotid artery plaques: 18F-fluoro-2-deoxyglucose positron emission tomography, computed tomography, and magnetic resonance imaging. *Stroke*. 2009;40(12):3718-3724.

(25) Rudd JH, Myers KS, Bansilal S et al. Relationships among regional arterial inflammation, calcification, risk factors, and biomarkers: a prospective

fluorodeoxyglucose positron-emission tomography/computed tomography imaging study. *Circ Cardiovasc Imaging*. 2009;2(2):107-115.

(26) Rominger A, Saam T, Wolpers S et al. 18F-FDG PET/CT identifies patients at risk for future vascular events in an otherwise asymptomatic cohort with neoplastic disease. *J Nucl Med*. 2009;50(10):1611-1620.

(27) Menezes LJ, Kotze CW, Hutton BF et al. Vascular inflammation imaging with 18F-FDG PET/CT: when to image? *J Nucl Med*. 2009;50(6):854-857.

(28) Wasselius J, Larsson S, Jacobsson H. Time-to-time correlation of high-risk atherosclerotic lesions identified with [(18)F]-FDG-PET/CT. *Ann Nucl Med*. 2009;23(1):59-64.

(29) Wasselius JA, Larsson SA, Jacobsson H. FDG-accumulating atherosclerotic plaques identified with 18F-FDG-PET/CT in 141 patients. *Mol Imaging Biol*. 2009;11(6):455-459.

(30) Potter K, Lenzo N, Eikelboom JW, Arnolda LF, Beer C, Hankey GJ. Effect of long-term homocysteine reduction with B vitamins on arterial wall inflammation assessed by fluorodeoxyglucose positron emission tomography: a randomised double-blind, placebo-controlled trial. *Cerebrovasc Dis*. 2009;27(3):259-265.

(31) Graebe M, Pedersen SF, Borgwardt L, Hojgaard L, Sillesen H, Kjaer A. Molecular pathology in vulnerable carotid plaques: correlation with [18]-fluorodeoxyglucose positron emission tomography (FDG-PET). *Eur J Vasc Endovasc Surg*. 2009;37(6):714-721.

(32) Rudd JH, Myers KS, Bansilal S et al. Atherosclerosis inflammation imaging with 18F-FDG PET: carotid, iliac, and femoral uptake reproducibility, quantification methods, and recommendations. *J Nucl Med*. 2008;49(6):871-878.

(33) Paulmier B, Duet M, Khayat R et al. Arterial wall uptake of fluorodeoxyglucose on PET imaging in stable cancer disease patients indicates higher risk for cardiovascular events. *J Nucl Cardiol*. 2008;15(2):209-217.

(34) Bural GG, Torigian DA, Chamroonrat W et al. FDG-PET is an effective imaging modality to detect and quantify age-related atherosclerosis in large arteries. *Eur J Nucl Med Mol Imaging*. 2008;35(3):562-569.

(35) Arauz A, Hoyos L, Zenteno M, Mendoza R, Alexanderson E. Carotid plaque inflammation detected by 18F-fluorodeoxyglucose-positron emission tomography. Pilot study. *Clin Neurol Neurosurg*. 2007;109(5):409-412.

(36) Rudd JH, Myers KS, Bansilal S et al. (18)Fluorodeoxyglucose positron emission tomography imaging of atherosclerotic plaque inflammation is highly reproducible: implications for atherosclerosis therapy trials. *J Am Coll Cardiol*. 2007;50(9):892-896.

(37) Tahara N, Kai H, Nakaura H et al. The prevalence of inflammation in carotid atherosclerosis: analysis with fluorodeoxyglucose-positron emission tomography. *Eur Heart J*. 2007;28(18):2243-2248.

(38) Tahara N, Kai H, Yamagishi S et al. Vascular inflammation evaluated by [18F]-fluorodeoxyglucose positron emission tomography is associated with the metabolic syndrome. *J Am Coll Cardiol*. 2007;49(14):1533-1539.

(39) Wu YW, Kao HL, Chen MF et al. Characterization of plaques using 18F-FDG PET/CT in patients with carotid atherosclerosis and correlation with matrix metalloproteinase-1. *J Nucl Med*. 2007;48(2):227-233.

(40) Tahara N, Kai H, Ishibashi M et al. Simvastatin attenuates plaque inflammation: evaluation by fluorodeoxyglucose positron emission tomography. *J Am Coll Cardiol*. 2006;48(9):1825-1831.

(41) Tawakol A, Migrino RQ, Bashian GG et al. In vivo 18F-fluorodeoxyglucose positron emission tomography imaging provides a noninvasive measure of carotid plaque inflammation in patients. *J Am Coll Cardiol*. 2006;48(9):1818-1824.

(42) Davies JR, Rudd JH, Fryer TD et al. Identification of culprit lesions after transient ischemic attack by combined 18F fluorodeoxyglucose positron-emission tomography and high-resolution magnetic resonance imaging. *Stroke*. 2005;36(12):2642-2647.

(43) Rudd JH, Warburton EA, Fryer TD et al. Imaging atherosclerotic plaque inflammation with [18F]-fluorodeoxyglucose positron emission tomography. *Circulation*. 2002;105(23):2708-2711.
