# Supplementary material for: Variability in quantitative analysis of atherosclerotic plaque inflammation using 18F-FDG PET/CT
Source: PLoS One. 2017 Aug 11;12(8):e0181847. doi: 10.1371/journal.pone.0181847 (PMC5553940; doi:10.1371/journal.pone.0181847)
Supplement: S2 Table — (PDF) [file pone.0181847.s007.pdf]

Circular ROI's were drawn on axial CT-images covering vessel wall and blood pool. Sagittal and coronal slices were used to verify correct ROI-positioning.

**Blood vessel**	
Right carotid	From where right common carotid artery branches from brachiocephalic trunc until right carotid bifurcation
Left carotid	Proximal part of aortic arch from which left common carotid branches until left carotid bifurcation
Aortic arch	From proximal branches until distal part of ascending aorta and proximal part of descending aorta
Ascending aorta	First slice proximal to aortic arch region until 1 slice above aortic root
Descending aorta	First slice distal to aortic arch region, 15 slices downwards
Abdominal aorta	From 1 slice distal to renal artery branches until abdominal aorta bifurcation
Right iliac	1 slice distal from aortic bifurcation until bifurcation right common iliac artery
Left iliac	1 slice distal from aortic bifurcation until bifurcation left common iliac artery
Right femoral	10 slices downward from inguinal ligament
Left femoral	10 slices downward from inguinal ligament

Table 2. Predefined methodology for drawing regions of interest (ROI's) on axial CT-slices
